# Androgen receptor uses relaxed response element stringency for selective chromatin binding and transcriptional regulation *in vivo*

**DOI:** 10.1093/nar/gkt1401

**Published:** 2014-01-22

**Authors:** Biswajyoti Sahu, Päivi Pihlajamaa, Vanessa Dubois, Stefanie Kerkhofs, Frank Claessens, Olli A. Jänne

**Affiliations:** ^1^Department of Physiology, Institute of Biomedicine, Biomedicum Helsinki, University of Helsinki, FI-00014 Helsinki, Finland and ^2^Department of Cellular and Molecular Medicine, Molecular Endocrinology Laboratory, Katholieke Universiteit Leuven, Campus Gasthuisberg, BE-3000 Leuven, Belgium

## Abstract

The DNA-binding domains (DBDs) of class I steroid receptors—androgen, glucocorticoid, progesterone and mineralocorticoid receptors—recognize a similar *cis*-element, an inverted repeat of 5′-AGAACA-3′ with a 3-nt spacer. However, these receptors regulate transcription programs that are largely receptor-specific. To address the role of the DBD in and of itself in ensuring specificity of androgen receptor (AR) binding to chromatin *in vivo*, we used SPARKI knock-in mice whose AR DBD has the second zinc finger replaced by that of the glucocorticoid receptor. Comparison of AR-binding events in epididymides and prostates of wild-type (wt) and SPARKI mice revealed that AR achieves selective chromatin binding through a less stringent sequence requirement for the 3′-hexamer. In particular, a T at position 12 in the second hexamer is dispensable for wt AR but mandatory for SPARKI AR binding, and only a G at position 11 is highly conserved among wt AR-preferred response elements. Genome-wide AR-binding events agree with the respective transcriptome profiles, in that attenuated AR binding in SPARKI mouse epididymis correlates with blunted androgen response *in vivo*. Collectively, AR-selective actions *in vivo* rely on relaxed rather than increased stringency of *cis*-elements on chromatin. These elements are, in turn, poorly recognized by other class I steroid receptors.

## INTRODUCTION

Androgens mediate the effects of male sex steroids through the androgen receptor (AR) that regulates expression of androgen-dependent genes by binding to *cis*-elements in the regulatory regions of target genes. The DNA-binding domain (DBD) of class I steroid receptors—AR, glucocorticoid receptor (GR), progesterone receptor (PR) and mineralocorticoid receptor (MR)—is highly conserved. They all recognize a similar palindromic response element that comprises an inverted repeat of the 5′-AGAACA-3′ hexamer with a 3-nt spacer, usually termed as a canonical androgen/glucocorticoid response element (ARE/GRE) (1). The first hexamer is highly conserved and represents the high-affinity site for receptor interaction because mutations in this core, as opposed to the second one, have a strong impact on receptor binding and reporter gene responsiveness, as shown by *in vitro* assays (2). In transient transfection experiments, AR, GR, PR and MR are all capable of binding to the canonical ARE/GRE and inducing expression of reporter genes driven by the canonical sequence (3). Moreover, genome-wide analyses using chromatin immunoprecipitation (ChIP) coupled with high-throughput sequencing (ChIP-seq) experiments have shown that AR and GR bind to shared loci on native chromatin (4–6). One-half of the AR cistrome overlaps with that of GR in LNCaP-1F5 prostate cancer cells, and ligand-occupied GR is able to modulate the AR pathway (5). These results present a biological enigma: how are specific responses to cognate ligands of class I steroid receptors ensured in cells that express multiple receptors at the same time.

One potential explanation for specific responses to class I steroid receptor ligands is subtle differences in the *cis*-elements that receptor DBDs recognize on chromatin. Selective AREs resembling direct repeats of the 5′-AGAACA-3′ hexamer are not recognized by GR or MR *in vitro* (3,7,8). A chimeric AR, in which the second zinc finger of the DBD is replaced by that of GR, possesses significantly attenuated affinity for selective AREs in transient transfection experiments and *in vitro* binding assays, whereas transactivation by the chimeric receptor *via* the classical ARE/GREs is similar to or better than that by wild-type (wt) AR (9). A transgenic mouse line with the same AR to GR switch in the second zinc finger of the DBD, dubbed SPARKI (specificity-affecting AR knock-in) mice, has smaller reproductive organs and reduced fertility compared with wt littermates, although androgen-dependent anabolic parameters, such as body weight and muscle strength, are unaffected (9). SPARKI males show reduced sperm count and impaired sperm maturation, suggesting defective function of the epididymis, a highly androgen-responsive reproductive organ (10).

SPARKI mice provide an excellent *in vivo* model system to investigate the importance of DBD structure in and of itself in setting apart genome-wide androgen-specific responses from those potentially regulated by glucocorticoids as well. Here, we compared AR-binding events and transcription programs in epididymides and prostates of wt and SPARKI male mice to delineate potential genome-wide rules behind androgen-selective responses. Our results show that, counter-intuitively, AR selectivity is achieved by relaxed rather than tightened *cis*-element stringency at chromatin binding sites which, in turn, attenuates binding of other class I steroid receptors to these *cis*-elements.

## MATERIALS AND METHODS

### Mice and hormone treatments

All the experiments were carried out using 12-week-old adult male mice of C57BL/6 strain. SPARKI knock-in mice have been described previously (9). Wt littermates were used as controls. For ChIP assays, caput epididymides and ventral prostates were collected from intact wt and SPARKI male mice. For mRNA isolation, caput epididymides were harvested from castrated wt and SPARKI mice. Testosterone treatment was brought about by sc Silastic implants (Silclear Tubing; Degania Silicone, Jordan Valley, Israel) for 7 days, corresponding to a daily dose of 23 µg. Control group received empty implants. All experimental work involving animals was conducted with approval of the Katholieke Universiteit Leuven ethical committee.

### Chromatin immunoprecipitation

ChIP assays were performed as described previously (4) with modification for tissues (11). Ventral prostates from four and caput epididymides from six wt mice and eight SPARKI mice were pooled to yield one 500 -µl chromatin sample. Frozen tissues were pulverized and cross-linked with 1% formaldehyde for 20 min at room temperature. Formaldehyde was quenched by adding 0.125 M glycine for 5 min at room temperature, followed by two washes with ice-cold phosphate-buffered saline. The cross-linked tissues were homogenized in hexylene glycol buffer (1 M hexylene glycol, 0.1 mM MgCl_2_, 5 mM ethylene glycol tetraacetic acid, 1 mM PIPES, 2 mM dithiothreitol, 1 × proteinase inhibitor cocktail; Roche Diagnostics, Indianapolis, IN, USA) using Ultra Turrax (Ika, Staufen, Germany), filtered through nylon net (20 µm), and nuclei isolated by low-speed centrifugation. The crude nuclear pellet was resuspended in radioimmunoprecipitation assay buffer and sonicated using a micro-tip sonicator (Misonix Inc., Farmingdale, NY, USA) to yield chromatin fragments of 100–500 bp in size. Anti-AR (12) and normal rabbit IgG (sc-2027, Santa Cruz, Dallas, TX, USA) antibodies were conjugated to Dynabeads (Invitrogen, Carlsbad, CA, USA) and incubated with sonicated chromatin overnight at 4°C, followed by five washes in LiCl buffer and incubation at 65°C overnight for reverse crosslinking. Immunoprecipitated DNA was purified and used for quantitative polymerase chain reaction (qPCR) analysis. In ChIP–qPCR, immunoprecipitated and input DNA were amplified using SYBR Green Mastermix (Roche) and specific primers (Supplementary Table S1), and the results are shown as percentage of input values.

### ChIP-seq library preparation

ChIP samples were processed according to Illumina’s library preparation protocol by pooling three immunoprecipitates for each library (4). In brief, DNA samples were blunt-ended, A-tailed and ligated to sequencing adapters followed by size selection (size range 150–300 bp) on agarose gel. Excised fragments were purified using Qiaquick Gel Extraction kit (Qiagen GmbH, Hilden, Germany) followed by PCR amplification for 20 cycles. The purified DNA was sequenced using GAII (Illumina Inc., San Diego, CA, USA) at Biomedicum Functional Genomics core facility. ChIP-seq reads were filtered according to Illumina’s instructions using the Illumina chastity filter, and reads were aligned to mouse genome (mm9) using Bowtie without any mismatches. ChIP-seq experiments were carried out in biological duplicates.

### Bioinformatics analysis

ChIP-seq peak calling and differential peak calling were performed using MACS2 (13,14) and HOMER (15) algorithms. The overlap analysis, tag density maps and binding site correlation plots were performed using Cistrome (16). The *de novo* motif analysis was performed using MEME Suite (17) and HOMER motif discovery algorithm (15). Data visualization was carried out using Integrative Genomics Viewer (18). Gene expression profiles for differentially expressed genes in whole epididymis of intact wt and SPARKI mice (10) were compared with the differentially androgen-regulated genes in caput epididymis (11).

### Electrophoretic mobility shift assay

AR and GR DBD fragments of ∼11 kDa in size, corresponding to residues 533–637 of rat AR DBD and residues 432–533 of rat GR DBD, were expressed as glutathione S-transferase fusion proteins in the *Escherichia coli* BL21 strain (8). The GST tag was removed by thrombin cleavage. Double-stranded oligonucleotides containing ARE sequences (Supplementary Table S1) were labeled with [α-^32^P]dCTP by a filling-in with the Klenow fragment of DNA polymerase. Slp-MUT and Slp-HRE sequences have been described (3). Purified AR DBD or GR DBD (100 ng protein) was incubated with radiolabeled probe (20 000 cpm) in 10 mM HEPES (pH 7.9), 2.5 mM MgCl_2_, 0.05 mM ethylenediaminetetraacetic acid, 8% glycerol, 50 mM NaCl, 2.5 ng/μl poly (dIdC), 1 mM dithiothreitol and 0.05% Triton X-100 for 20 min on ice. DBD-bound and unbound probes were separated by electrophoresis on a 4% polyacrylamide gel. Radioactive bands were visualized with STORM 840 PhosphorImager (Molecular Dynamics).

### Transactivation assays

HeLa cells (ATCC, Manassas, VA, USA) were cultured in Dulbecco's modified Eagle's medium containing 4.5 g/l glucose, 4 mM l-glutamine, penicillin (100 IU/ml)–streptomycin (100 μg/ml) (Sigma-Aldrich, St. Louis, MO, USA) and 10% fetal calf serum (Invitrogen). For transactivation experiments, cells were seeded onto 96-well plates (8000 cells/well) in Dulbecco's modified Eagle's medium supplemented with 5% charcoal-stripped serum (Sigma-Aldrich) and transfected using GeneJuice (Novagen, Merck KGaA, Darmstadt, Germany) with 10 ng of AR or GR expression plasmid (19), 100 ng of luciferase reporter plasmid containing four copies of the ARE of interest (Supplementary Table S1) and 5 ng of β-galactosidase expression plasmid (Stratagene, Santa Clara, CA, USA). Reporter plasmids were generated as previously described (3). Cells were lysed after a 24-h exposure to 10 nM methyltrienolone (Perkin Elmer, Waltham, MA, USA) or 10 nM dexamethasone (Sigma-Aldrich). Luciferase and β-galactosidase activities were measured, and transactivation activity was calculated (20).

### RNA isolation and quantitative reverse transcription PCR

Caput epididymides of wt and SPARKI mice (*n* = 5 in both cases) were harvested in RNALater (Qiagen) after a 7-day treatment with testosterone or vehicle. Total RNA was isolated using RNeasy Mini Kit (Qiagen). cDNA synthesis was carried out from 2 µg of total RNA with Transcriptor High Fidelity cDNA synthesis kit using random hexamers (Roche). Quantitative reverse transcription PCR (qRT-PCR) was performed using SYBR Green mastermix (Roche) and normalized to 18 S rRNA levels. Student’s *t*-test (*P* < 0.001) was used to calculate the statistical significance of differences in gene expression in wt and SPARKI mice between testosterone- and vehicle-treated groups and between testosterone-treated wt and SPARKI groups. The primer sequences are provided in Supplementary Table S1.

## RESULTS

### Genome-wide AR-binding events in wt and SPARKI epididymis

**Figure 1.**
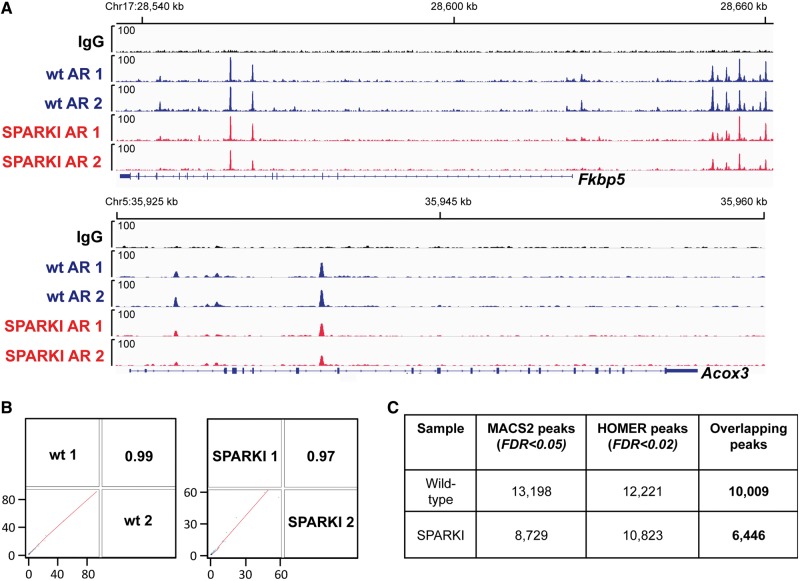
*In vivo* AR binding in epididymis of wt and SPARKI mice. (**A**) AR-binding events mapped to *Fkbp5* and *Acox3* loci in caput epididymis of wt and SPARKI male mice. ChIP-seq tracks from two independent biological replicates and non-specific rabbit IgG control are shown. (**B**) Genome-scale wiggle file correlation plot and correlation coefficient values for AR-binding events of two independent biological replicates in epididymides of wt and SPARKI mice. (**C**) The number of AR-binding peaks derived from concatenated biological replicates and using MACS2 and HOMER peak calling algorithms. (FDR, false discovery rate).

ChIP-seq analyses of *in vivo* AR-binding sites (ARBs) on epididymal chromatin were performed in biological duplicate samples. Examples of AR ChIP-seq tracks of wt and SPARKI mice are shown in Figure 1A at two genomic loci that are androgen-responsive in murine epididymis (11); *Fkbp5* (FK506 binding protein 5) encoding a chaperone protein and *Acox3* (acyl-coenzyme A oxidase 3) involved in fatty acid metabolism in peroxisomes. AR occupancy is indistinguishable at the *Fkbp5* regulatory region between wt and SPARKI mice, which agrees with the result that *Fkbp5* mRNA accumulation is induced by androgen to the same extent in epididymides of these mice (10). Sequenced reads were aligned to mouse genome (mm9) using bowtie, and peak calling for individual replicates was carried out using MACS2 (13,14). Importantly, the biological replicates show excellent concordance, as judged by individual ChIP-seq tracks (Figure 1A) and by coefficient values from correlation analyses of the raw sequence data (Figure 1B). To increase the depth of analysis, we concatenated independent biological replicates and re-performed peak calling using two different algorithms, MACS2 and HOMER (15). The peaks called from the two algorithms were used to compute the final overlapping peaks (with at least 1 nt overlap), resulting in AR cistromes comprising 10 009 and 6446 ARBs in wt and SPARKI epididymides, respectively (Figure 1C, Supplementary Data sets S1 and S2).

### SPARKI AR fails to recognize wt AR-preferred enhancers

**Figure 2.**
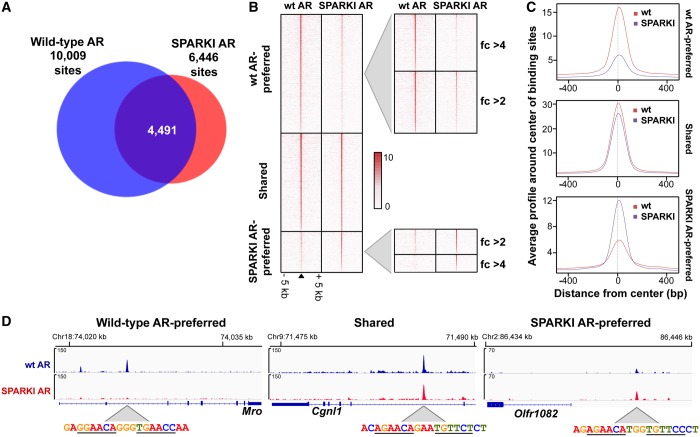
Features of the AR-binding events in epididymides of wt and SPARKI mice. (**A**) Area-proportional Venn diagrams of AR cistromes in epididymis of wt and SPARKI mice. (**B**) Tag density maps of AR-binding events in wt and SPARKI epididymides at wt AR-preferred, shared and SPARKI AR-preferred binding sites in a span of ±5 kb. Tag density maps of AR-binding events in wt and SPARKI epididymides at wt AR-preferred sites showing 2–4-fold or >4-fold difference in the differential enrichment analysis (wt versus SPARKI), and at SPARKI AR-preferred sites showing 2–4-fold or >4-fold difference in SPARKI versus wt differential enrichment analysis using HOMER. (**C**) Average tag numbers of ARBs in a window of ±500 bp centered around the summit of the receptor-binding site in the three AR-binding event categories: wt AR-preferred, shared and SPARKI AR-preferred. (**D**) ChIP-seq track examples of AR-binding events in the three categories and the corresponding DNA sequence at the summit of the ARBs. Fold-enrichment ratios computed from ChIP-seq peaks: *Mro* (wt versus SPARKI) 78-fold, *Cgnl1* (wt versus SPARKI) 1.09-fold, *Olfr1082* (SPARKI versus wt) 3.3-fold.

Two-thirds of the SPARKI AR cistrome overlap with that of wt mice (Figure 2A). The greater number of ARBs that are unique to wt epididymis is explainable by the ability of wt AR to recognize selective AREs. Because the SPARKI AR is anticipated to recognize the canonical ARE/GRE only, it is intriguing that there are also *in vivo* AR-binding events that are favored by the SPARKI receptor (Figure 2A). As ChIP-seq provides a quantitative assessment of receptor binding across the genome, the tag density of AR binding to a given site could be used as a surrogate measure for the affinity of the receptor to this site. The tag density maps of AR-binding events and average tag profiles in wt and SPARKI mice indicate that the two receptors are loaded to the same extent onto sites shared by wt AR and SPARKI AR, most likely reflecting indistinguishable binding affinities of the receptors for these sites (Figure 2B and C). Overall, wt and SPARKI ARs bind to the shared sites with ∼2-fold higher affinity than to non-shared sites (Figure 2C). Interestingly, shared sites have also been reported to be the high-affinity binding loci for the estrogen receptor, when chromatin binding events in multiple estrogen target tissues or cell lines were compared with those with strict tissue- or cell-type specificity (21).

The ‘uniqueness’ of wt and SPARKI AR-binding events is relative rather than absolute, in that there are significant quantitative differences in the recruitment of the two ARs to these sites. Therefore, we call the two ARB groups as wt AR-preferred and SPARKI AR-preferred sites (Figure 2B and C). The tag density maps constructed for the wt AR-preferred sites with a >4-fold and a 2–4-fold change over SPARKI ARBs indicate that wt AR-binding events with high fold changes are poorly recognized by SPARKI AR (Figure 2B). The reverse is true for the AR-binding events preferred by SPARKI AR; these sites are recognized less well by wt AR (Figure 2B). The average tag profiles—a more quantitative assessment of the differences—reveal that the ARBs preferred by wt AR have tag numbers three times higher than those of SPARKI AR at the same sites, and the binding events preferred by SPARKI AR have twice the number of tags over those of wt AR at the same sites (Figure 2C). Collectively, these data indicate that there are classes of *cis*-elements that wt and SPARKI ARs recognize with dissimilar binding affinities.

Examples of ChIP-seq tracks are shown in Figure 2D with sequences at the summit of a shared AR-binding event (5′-acAGAACAgaaTGTTCTct-3′) and a SPARKI AR-preferred site (5′-agAGAACAtggTGTTCCct-3′). Both possess a canonical ARE/GRE, a palindrome of 5′-AGAACA-3′ with a 3-nt spacer. The example of a *cis*-element preferred by wt AR is a non-canonical ARE (Figure 2D) that bears resemblance to an imperfect direct repeat sequence (5′-gaGGAACAgggTGAACCaa-3′).

### WT AR selectivity *in vivo* depends on relaxed *cis*-element stringency

**Figure 3.**
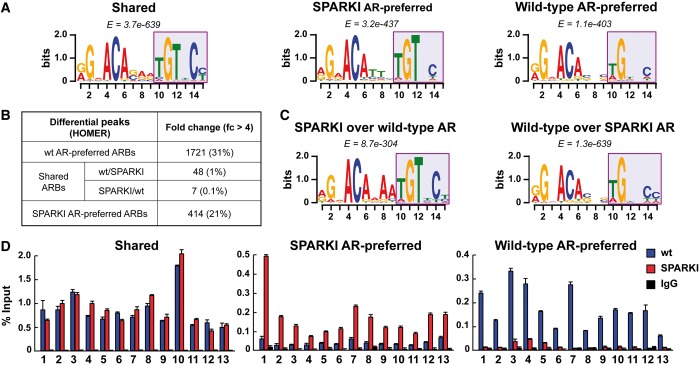
Sequence enrichment analysis and validation of the AR-binding events. (**A**) *Cis*-elements identified by *de novo* motif search for the ARBs belonging to shared, SPARKI AR-preferred and wt AR-preferred categories. (**B**) Differential enrichment analysis for wt versus SPARKI and SPARKI versus wt binding data sets by HOMER for the three categories of AR-binding events. The number of ARBs with differential enrichment >4 and the corresponding percentage of all ARBs in each category are shown. (**C**) *Cis*-elements enriched by *de novo* motif analysis for the binding peaks differentially enriched in SPARKI AR versus wt AR and in wt AR versus SPARKI AR. (**D**) Validation of AR-binding events by direct ChIP–qPCR assays for the three categories. ChIP was performed from chromatin samples obtained after pooling epididymides from six wt and eight SPARKI mice. Mean + SEM for two biological replicates are shown. In each category, 13 loci were selected for the analysis.

We hypothesized that genome-wide AR-binding events in epididymides of wt and SPARKI mice will help in establishment of defined rules for the *cis*-elements that AR recognizes on chromatin. To this end, we performed *de novo* motif search analyses using MEME Suite (17) for the three ARB categories: (i) binding sites shared by the two receptors or (ii) preferred by wt AR or (iii) SPARKI AR—on sequences comprising the top 1000 sites spanning ±15 bp around the summit of the receptor binding locus. As expected, an inverted palindromic repeat with a 3-nt gap resembling the canonical ARE/GRE is highly enriched among the shared ARBs (*E* = 3.7e-639). Interestingly, a similar *cis*-element is also highly enriched among the ARBs preferred by SPARKI AR (*E* = 3.2e-437). In both instances, there is a high degree of sequence conservation. The best conserved nucleotides are a G at position 2 and a C at position 5, followed by an A at positions 4 and 6, and their complementary partners T, G, T and C at positions 10, 11, 12 and 14, respectively (Figure 3A).

*De novo* motif search analyses of the binding sites preferred by wt AR uncovered a different *cis*-element (*E* = 1.1e-403), in which the 5′ hexamer is almost identical with that described above but separated by a 3-nt gap from a hexamer with weak sequence conservation (Figure 3A). This latter hexamer has a highly conserved G at position 11, along with weak conservation for T and C at positions 10 and 14, respectively. Importantly, the highly conserved T at position 12 of the canonical ARE/GRE present at the shared and SPARKI AR-preferred sites is dispensable among the *cis*-elements for wt AR binding. Thus, relaxed stringency in the *cis*-element sequence—dismissal of the conserved T at position 12 in particular—ensures selective wt AR binding to chromatin. This result agrees with a previous mutational analysis showing that substitution of an A with a T at this position in a selective ARE enhances GR binding and glucocorticoid response (2).

Wt AR-preferred and SPARKI AR-preferred binding events were next cross-compared directly by HOMER. This analysis revealed that 31% of the wt AR-preferred peaks have >4-fold enrichment over those to SPARKI AR (1721 ARBs, Supplementary Data set S3), and 21% of the SPARKI AR-preferred peaks have >4-fold enrichment over those of wt AR (414 ARBs, Supplementary Data set S4) (Figure 3B). Importantly, <1% of the shared AR-binding peaks show >4-fold differential enrichment, implying an equal binding affinity for wt and SPARKI AR at the shared sites (Figure 3B). To characterize further the nature of the *cis*-elements among the wt AR-preferred ARBs, *de novo* motif search analysis was performed on the top 500 ARBs with >4-fold difference between wt and SPARKI AR binding peaks (Figure 3B, uppermost and lowest lines). This analysis yielded binding motifs for wt and SPARKI AR that are similar to those obtained by direct *de novo* motif searches (*cf.* Figure 3A and C). Of note, different analysis parameters, such as different windows around the peak summits, the use of shuffled instead of top-enriched sites or different algorithms (HOMER or MEME Suite) did not change the results of *de novo* analysis. Direct ChIP–qPCR validation of 13 randomly chosen loci corresponding to AR-binding events in the three categories confirm that there are significant quantitative differences in the loading of AR onto sites preferred either by wt AR or SPARKI AR (Figure 3D).

### *In vitro* validation of the wt AR-preferred, SPARKI AR-preferred and shared *cis*-elements

**Figure 4.**
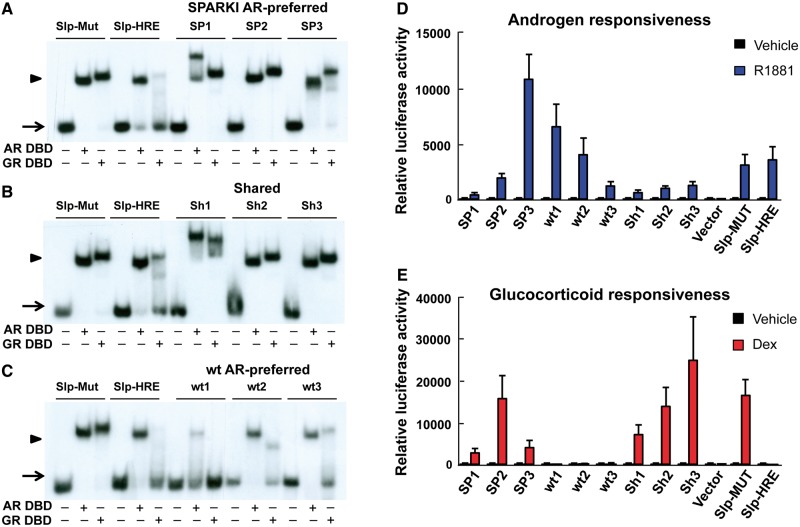
Validation of the three ARB categories by EMSAs and transactivation assays. Radiolabeled double-stranded oligonucleotides containing the AREs were tested for binding by purified AR DBD or GR DBD. Arrows refer to free probe and arrowheads to ARE DBD complexes. Slp-MUT and Slp-HRE are classical and selective ARE controls, respectively. (**A**) AREs from three SPARKI AR-preferred binding sites. (**B**) AREs from three shared binding sites. (**C**) AREs from three AR-preferred binding sites. HeLa cells were transiently transfected with AR (**D**) or GR (**E**) expression vector together with luciferase reporter constructs containing four copies of the putative AREs. Cells were exposed to methyltrienolone (R1881) (**D**) or dexamethasone (Dex) (**E**) for 24 h. Results are normalized to β-galactosidase activities and presented as mean + SEM values of three biological replicates performed in triplicate.

Electrophoretic mobility shift assay (EMSA) with AR DBD and GR DBD was used to examine the specificity of a few ARE sequences in the three categories under *in vitro* conditions. Classical and selective AREs, characterized in previous work (3), were used as controls. The second zinc finger of SPARKI AR is identical with that of GR, and SPARKI AR-preferred and shared sequences were bound by both AR DBD and GR DBD (Figure 4A and B). The AREs classified by our ChIP-seq analysis as wt AR-preferred sequences were not recognized by GR DBD at all or with low affinity (Figure 4C). Thus, the results from EMSAs using naked DNA and AR DBD or GR DBD agree with those of ChIP-seq experiments carried out in a genuine chromatin environment and with full-length receptors.

To validate the ARB categories further, three AREs from each group were cloned in four copies upstream of the luciferase reporter gene and exposed in transactivation assays in HeLa cells to methyltrienolone and dexamethasone for AR and GR, respectively. AR was able to transactivate *via* all nine AREs, confirming their functional enhancer nature (Figure 4D). Importantly, GR could transactivate only through shared and SPARKI-preferred ARBs comprising canonical AREs but not through wt AR-preferred ARE sequences (Figure 4E).

### Differential AR-binding events are linked to differentially expressed genes in wt and SPARKI epididymis

**Figure 5.**
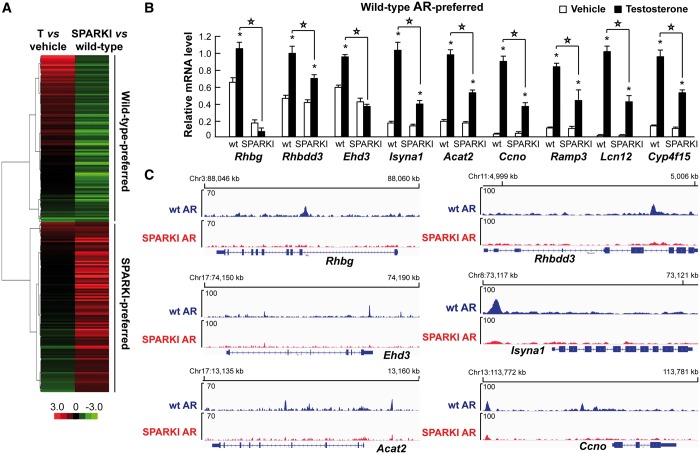
Comparison of AR-binding events and transcriptome profiles in wt and SPARKI epididymis. (**A**) Unsupervised hierarchical clustering of transcripts that are regulated by testosterone in caput epididymis (T versus vehicle) and expressed differentially in epididymides of intact SPARKI and wt male mice (SPARKI versus wt). (**B**) Relative mRNA levels, as measured by qRT-PCR, for wt-preferred androgen-regulated genes in epididymides of castrated wt or SPARKI mice treated with testosterone or vehicle. (Mean + SEM values are shown, *n* = 5). Statistically significant differences (*P* < 0.001) between testosterone-treated wt and SPARKI mice are indicated by *stars above the lines*, and statistically significant differences (*P* < 0.001) between vehicle- and testosterone-treated groups are depicted by *asterisks above the black bar* (testosterone) adjacent to the corresponding white bar (vehicle). (**C**) ChIP-seq tracks of six representative examples showing differences in AR loading onto genomic loci adjacent to AR-regulated genes in wt and SPARKI epididymides.

**Figure 6.**
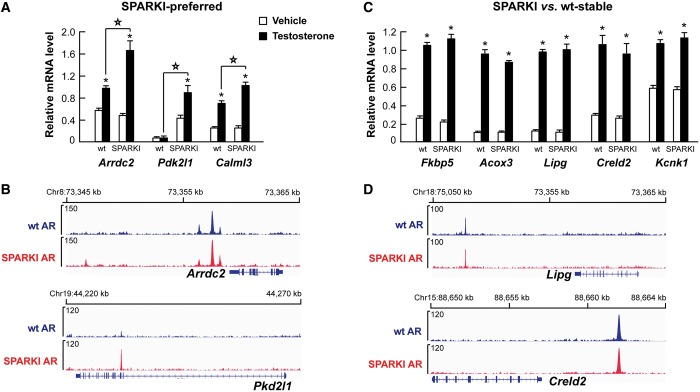
AR-binding events and androgen-regulated genes of wt and SPARKI epididymis for SPARKI-preferred genes and stably expressed genes in SPARKI versus wt conditions. (**A**) Relative mRNA levels, as measured by qRT-PCR, for SPARKI-preferred androgen-regulated genes in wt and SPARKI epididymides of castrated male mice treated with vehicle or testosterone. (Mean + SEM values, *n* = 5). Statistical significant differences (*P* < 0.001) between wt and SPARKI mice after androgen exposure (testosterone) are indicated by *stars above the lines*, and statistically significant differences (*P* < 0.001) between testosterone- and vehicle-treated wt or SPARKI mice are depicted by *asterisks above the black bar* (testosterone) adjacent to the corresponding white bar (vehicle). (**B**) ChIP-seq tracks illustrating AR binding at genomic loci adjacent to the androgen-regulated *Arrdc2* and *Pkd2l1* genes in wt and SPARKI epididymis. (**C**) Relative mRNA levels, as measured by qRT-PCR, for androgen-regulated genes expressed to the same level (stable) in epididymides of intact wt and SPARKI mice. Castrated male mice were treated with testosterone or vehicle for 3 days. (Mean + SEM values, *n* = 5). Statistical significant differences (*P* < 0.001) between testosterone- and vehicle-treated groups are depicted by *asterisks above the black bar* (testosterone) adjacent to the corresponding white bar (vehicle). (**D**) ChIP-seq tracks showing AR loading onto genomic loci adjacent to the androgen-regulated *Lipg* and *Creld2* genes in wt and SPARKI epididymides.

By examining the gene expression data from whole epididymides of intact wt and SPARKI male mice (10), we found 511 transcripts with higher expression in wt than SPARKI mice (= wt-preferred genes) and 843 transcripts with higher expression in SPARKI than wt mice (= SPARKI-preferred genes) (fold change ≥1.5, *P* < 0.05). Even though intact male mice have physiological androgen levels, these differentially expressed transcripts may not all be androgen-regulated. To address this issue, we compared the Kerkhofs *et al.* data set (10) with androgen-regulated genes in caput epididymides of castrated male mice treated for 3 days with testosterone or vehicle (11) and identified 219 androgen-regulated transcripts that are differentially expressed in intact SPARKI versus wt mice (110 androgen upregulated and 109 androgen downregulated transcripts, Supplementary Data set S5). Unsupervised hierarchical clustering of the 219 transcripts revealed that most of the wt-preferred genes are upregulated by androgen, whereas most of the SPARKI-preferred genes are downregulated by androgen exposure (Figure 5A). Importantly, the wt-preferred androgen-regulated genes such as *Rhbg*, *Rhbdd3*, *Ehd3*, *Isyna1*, *Acat2*, *Ccno, Ramp3, Lcn12* and *Cyp4f15* show blunted upregulation by testosterone treatment in SPARKI epididymis compared with wt, as judged by mRNA accumulation (Figure 5B). Moreover, loss or attenuation of AR binding at regulatory sites of the wt-preferred genes *Rhbg*, *Rhbdd3*, *Ehd3*, *Isyna1*, *Acat2* and *Ccno* is observed in SPARKI epididymis compared with wt (Figure 5C). Likewise, qRT-PCR assays confirm the differential expression of SPARKI-preferred genes in SPARKI epididymis compared with the wt epididymis as seen in the case of *Arrdc2*, *Pdk2l1* and *Calml3* (Figure 6A). There is greater loading of SPARKI AR than wt AR onto the *Pkd2l1* locus, but in some instances, such as *Arrdc2*, there is no clear difference between wt AR and SPARKI AR-binding events (Figure 6B). Examples of androgen-regulated genes (*Fkbp5*, *Acox3*, *Lipg*, *Creld2* and *Kcnk1*) that are stably expressed in both wt and SPARKI epididymides are shown in Figure 6C. In agreement with transcript accumulation, AR loading is equal onto the respective regulatory regions in wt and SPARKI epididymis, as exemplified by *Fkbp5* and *Acox3* (Figure 1A) and *Lipg* and *Creld2* (Figure 6D).

### Relaxed AR *cis*-element stringency is not limited to the epididymis

**Figure 7.**
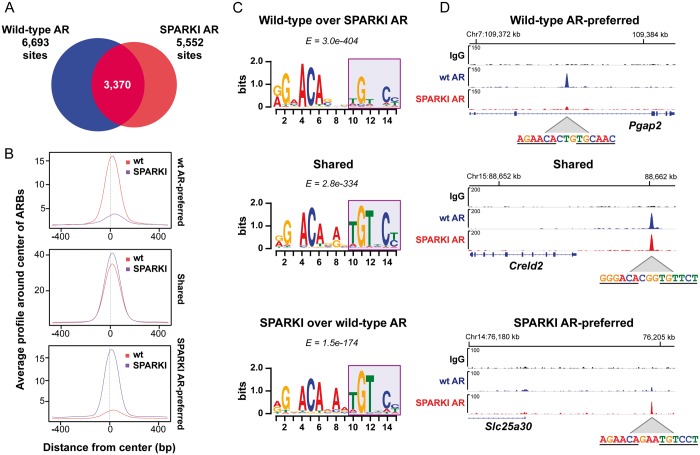
AR cistromes in wt and SPARKI prostates. (**A**) Area-proportional Venn diagrams of AR cistromes in prostates of wt and SPARKI mice. (**B**) Average tag numbers of ARBs in a window of ±500 bp centered around the summit of the receptor-binding site in the three AR-binding event categories: wt AR-preferred, shared and SPARKI AR-preferred. (**C**) *Cis*-elements revealed by *de novo* motif analyses of the top 500 binding peaks differentially enriched in SPARKI AR versus wt AR and in wt AR versus SPARKI AR and shared ARBs. (**D**) ChIP-seq track examples of AR-binding events in the three categories and the corresponding DNA sequence at the summit of the ARBs. Fold-enrichment ratios computed from ChIP-seq peaks: *Pgap2* (wt versus SPARKI) 13-fold, *Creld2* (wt versus SPARKI) 1.08-fold, *Slc25a30* (SPARKI versus wt) 4.8-fold.

To examine whether the finding on *in vivo* AR selectivity also applies to another androgen target tissue, we performed AR ChIP-seq on ventral prostates of SPARKI mice and their wt littermates. Peak calling with MACS2 and HOMER identified 6693 and 5552 high-confidence AR-binding events in prostates of wt and SPARKI mice, respectively, 3370 of which are shared by the two receptors (Figure 7A, Supplementary Data sets S6 and S7). SPARKI AR has low affinity to wt AR-preferred sites and wt AR to SPARKI AR-preferred sites, whereas both receptors bind to shared sites with high affinity (Figure 7B). Of the wt AR-preferred ARBs, 1079 sites have >4-fold enrichment over SPARKI AR (Supplementary Data set S8), and 609 SPARKI AR-preferred sites have >4-fold enrichment over wt AR (Supplementary Data set S9). Importantly and similar to epididymis, the *cis*-element identified by *de novo* motif search among the wt AR-preferred ARBs in prostate shows weak conservation of the second hexamer sequence, highlighting the relaxed *cis*-element stringency, with the G at position 11 being an important determinant for selective AR binding *in vivo* (Figure 7C). In a manner identical with that in epididymis, SPARKI AR-preferred and shared ARBs are enriched in prostate for the canonical ARE/GRE (Figure 7C). Examples of AR ChIP-seq tracks at three androgen-responsive loci in murine prostate, *Pgap2*, *Creld2* and *Slc25a30* (11) depict wt and SPARKI AR loading onto enhancers of the three ARB categories—wt and SPARKI AR-preferred or shared—and show the respective ARE sequences at peak summits (Figure 7D).

## DISCUSSION

Previous work has shown that there are two types of *cis-*elements that are recognized by the AR: (i) classical AREs corresponding to the canonical ARE/GRE and recognized by other class I steroid receptors as well, and (ii) selective AREs recognized primarily by AR and showing features that resemble direct repeats of the 5'-AGAACA-3' hexamer (3,10,22). By using genome-wide *in vivo* AR ChIP-seq on epididymal and prostatic chromatin of wt and SPARKI mice, we show in this work that the SPARKI AR fails to bind or binds with blunted affinity to sites corresponding to selective AREs. A significant proportion—55 and 50% of the epididymal and prostatic AR cistromes, respectively—comprised wt AR-preferred ARBs affirming the biological importance of selective AREs in ensuring specific AR responses. Surprisingly, the genome-wide analyses revealed that the selectivity of AR binding over that of other class I steroid receptors relies on relaxed rather than more stringent requirement for the response element sequence. Attenuated binding of SPARKI AR to selective AREs was, in turn, accompanied by blunted response to androgens of the corresponding genes in SPARKI mice.

The 5' hexameric core is almost identical between canonical ARE/GREs and selective AREs, and this hexamer is suggested to be the high-affinity DNA sequence for the binding of the first DBD (22,23). The contact of the first DBD to the high-affinity site is assumed to lead to a conformational change in the receptor protein, and possibly also in the DNA element, which subsequently facilitate binding of the second DBD to the less conserved low-affinity site (22,23). Previous work has indicated that the 3′ hexamer of selective AREs resembles, in many instances, an imperfect direct repeat of the 5′ hexameric sequence 5′-AGAACA-3′ [summarized in (22)]. Our current results show why this would be the case, in that the Gs at positions 2 and 11 are highly conserved in the selective AREs, permitting 3′ hexameric sequences that can be interpreted as being imperfect direct repeats. More importantly, however, selective AREs exhibit no requirement for a T at position 12. Genome-wide ChIP-seq experiments on several cell lines or tissues have shown that *cis*-elements for GR and PR contain a highly conserved T at this position (4–6,24–27). Thus, AR selectivity is achieved by loosening the structural stringency in the 3′ hexameric sequence, and consequently, other class I steroid receptors fail to bind to this element. Crystal structure studies have indicated that AR DBD has substitutions that create higher affinity for the receptor dimer interface than that of GR DBD, and subsequently, the AR could bind to a more diverse set of *cis*-elements with higher affinity and specificity than the GR (22,23). Thus, the high-affinity protein–protein binding at dimer interface rather than stringent *cis*-element structure is important for AR selectivity over the other class I type receptors. Other features in the receptor structure could possibly also contribute to the different binding affinities of AR and GR as well, as mutations changing the amino acids in the dimerization interface did not affect *in vitro* DNA-binding affinities or transactivation capabilities of the two receptors (28).

We examined in this work the role of DBD in and of itself in assuring the selectivity of AR signaling by comparing wt and chimeric receptors. However, specific transcriptional outcome *in vivo* by steroid receptors is influenced not only by the *cis*-element and its interaction with the DBD, but also by a number of chromatin modifications and divergent collaborating factors (29). Pioneer factors are proteins capable of binding to compact chromatin and priming it for steroid receptor binding. In prostate cancer cells and in normal mouse prostate, AR binding is primed by FoxA1, and a distinct *cis*-element, composed of an ARE half-site and a FoxA1 element, is highly enriched among the AR-binding sequences (4,11). Pioneer factors have been reported to guide lineage-, cell-type- and tissue-specific AR binding (5,11,30), and the interplay between steroid receptors and pioneer factors is likely to contribute to the specificity of steroid receptor binding in addition to the DNA–DBD interaction. However, we did not find markedly dissimilar compilations of *cis*-elements for other transcription factors adjacent to the three ARE categories that would explain their divergent receptor binding properties (Supplementary Data set S10).

The determinants of preferential *in vivo* binding of SPARKI AR to a subset of ARBs remain elusive. The DNA sequence in itself may act as an allosteric ligand for nuclear receptors, thereby affecting receptor conformation and activity (31). However, it is unlikely that this phenomenon is the main reason for the presence of SPARKI AR-preferred ARBs *in vivo*, as the SPARKI AR sites contain the same ARE sequence as the shared sites. Moreover, the *cis*-elements of SPARKI AR-preferred ARBs in isolation, i.e.*,* in EMSA experiments and reporter gene assays (*cf.* Figure 4), lost their selectivity and responded equally well to wt AR and GR, supporting the importance of local chromatin environment. Differences in local chromatin structure, such as histone and DNA modifications, could explain the occurrence of these sites (32). Finally, in addition to *cis*-elements, cross-talk of steroid receptors with tissue-specific pioneer factors and/or coregulators is likely to influence receptor recruitment to selected chromatin sites and determine the final transcriptional outcome.

## ACCESSION NUMBERS

The ChIP-seq data have been deposited in the Gene Expression Omnibus database with accession number GSE51106, and the gene expression data with accession number GSE47181.

## SUPPLEMENTARY DATA

Supplementary Data are available at NAR Online.

## FUNDING

Academy of Finland, Sigrid Jusélius Foundation, Finnish Cancer Foundations, Biocentrum Helsinki, Helsinki University Central Hospital, Helsinki Biomedical Graduate Program and research grant of the KU Leuven nr OT/11/081. Holder of a doctoral fellowship of the Fund for Scientific Research Flanders (FWO) (to V.D.). Funding for open access charge: Sigrid Jusélius Foundation and Biocentrum Helsinki.

*Conflict of interest statement.* None declared.

## Supplementary Material

Supplementary Data
