# Supplementary material for: Androgen receptor uses relaxed response element stringency for selective chromatin binding and transcriptional regulation in vivo
Source: Nucleic Acids Res. 2014 Jan 22;42(7):4230–40. doi: 10.1093/nar/gkt1401 (PMC3985627; doi:10.1093/nar/gkt1401)
Supplement: Supplementary Data [file supp_42_7_4230__index.html]

Androgen receptor uses relaxed response element stringency for selective chromatin binding and transcriptional regulation in vivo — Androgen receptor uses relaxed response element stringency for selective chromatin binding and transcriptional regulation in vivo — Supplementary Data 

# Androgen receptor uses relaxed response element stringency for selective chromatin binding and transcriptional regulation *in vivo*

## Supplementary Data

files

**Files in this Data Supplement:**

- Supplementary Data - pdf file
- Supplementary Data - xlsx file
