# Supplementary material for: Androgen receptor uses relaxed response element stringency for selective chromatin binding and transcriptional regulation in vivo
Source: Nucleic Acids Res. 2014 Jan 22;42(7):4230–40. doi: 10.1093/nar/gkt1401 (PMC3985627; doi:10.1093/nar/gkt1401)
Supplement: Supplementary Data [file supp_gkt1401_nar-03055-x-2013-File009.pdf]

Table S1. Sequences of the primers used for ChIP and qRT-PCR assays and ARE sequences used for EMSA and transfection assays.

**Primer name for ChIP primers**	**Primer sequence (5' → 3')**
**Shared ARBs**	
Shared-ARB1+	AGGAGTCACACCCTTCTGGA
Shared-ARB1-	GGAAAGAACCCAGGGAGGTA
Shared-ARB2+	ACCTCAGGTGCAGTGATGC
Shared-ARB2-	TGCTTGGAGATCTGTCATGG
Shared-ARB3+	CCAATGGAGAACGGAATGTT
Shared-ARB3-	GCCCCATTATCTTTTGTGTCTC
Shared-ARB4+	TGTCTGGCATGATTTCCTGA
Shared-ARB4-	CCACAGGGTTCTTGTCACCT
Shared-ARB5+	AAGGCCAAGTACACGGTCAC
Shared-ARB5-	ACGTGCCCAAAATAGCTCAC
Shared-ARB6+	CTTCTAAGGCTCCCCTTGCT
Shared-ARB6-	TCCCTTCAGCCCTGACAA
Shared-ARB7+	GTCAGGCACTAGGACCCAGA
Shared-ARB7-	CTGTGGGTGAGCTGAGAACA
Shared-ARB8+	GGTCAGGTTCAGGTTCAAGG
Shared-ARB8-	AGCGGAGAGGCAAAGTACAG
Shared-ARB9+	GCCAACACCTGTGAGTTCAA
Shared-ARB9-	CAGATTACTGGGGACAGAACG
Shared-ARB10+	CAGATTACTGGGGACAGAACG
Shared-ARB10-	CAGCATGTCCTGTCCTTGG
Shared-ARB11+	CTCCAGAGTGGTGGGTGTAGA
Shared-ARB11-	ATGATGTTCTGGGACCAAGC
Shared-ARB12+	GCTTGGCCTTGGTTAAAGTG
Shared-ARB12-	GCCCTGAAGCAACTTCCTC
Shared-ARB13+	AAAGCCTTCCTCCCTCAGAC
Shared-ARB13-	TGAGGACTCTCCCTGTGTGAT
**SPARKI AR-preferred sites**	
SPARKI-ARB1+	GCCAACCATCATCACCAGA
SPARKI-ARB1-	GCCCCATCTTGATTAATTGTGT
SPARKI-ARB2+	AATTGGTCTCAGGGGAGGAG
SPARKI-ARB2-	AATTGGTCTCAGGGGAGGAG
SPARKI-ARB3+	TGTCTTGTCTGGCCTCATTG

SPARKI-ARB3-	CATGGGTTCCAATTTGTCCT
SPARKI-ARB4+	TGGAGGAGAGGACATAGCAAA
SPARKI-ARB4-	GAGGATGCCTTTCACACCTC
SPARKI-ARB5+	GTGTCAGGTGTCCAGGAGGT
SPARKI-ARB5-	AAGCCATGCTCTAGCAGCTC
SPARKI-ARB6+	GGTCTTGTGACGAGCATCAA
SPARKI-ARB6-	AGGAAGAAGCCACTTGAAAGG
SPARKI-ARB7+	CACTGAGGGGCCAGTAACAT
SPARKI-ARB7-	ATATTGTCCGCTGGAAGCAT
SPARKI-ARB8+	TTGAGAACTGCTGCTCCAGA
SPARKI-ARB8-	TGCTGACCGCTTAGAAAACA
SPARKI-ARB9+	GCTATCCAGGTGGCTGTGTT
SPARKI-ARB9-	GCTGGAAAGAACGGAGTGTC
SPARKI-ARB10+	GTTCCGTATCCCAACTGCAT
SPARKI-ARB1+-	GTCCATGGCTCAGGGAACT
SPARKI-ARB11+	TGCAGTGTCGAGAACATTTTG
SPARKI-ARB11-	CTGCCTGAAGAACCAGGGTA
SPARKI-ARB12+	TTGCGCAGGAACACAAAGT
SPARKI-ARB12-	GCAGTTGTTGGGCAGATGT
SPARKI-ARB13+	GTATGGCCACTTGGCTTCAT
SPARKI-ARB13-	TGATCGGGAACAAGACACAA
**Wild-type AR-preferred sites**	
WT-ARB1+	AGTACGGGCTCATTCCACAC
WT-ARB1-	CATGCTGTGGCTCATTCTTG
WT-ARB2+	CAGTGGCAAAGATGGACAGA
WT-ARB2-	ACAGCGTGAAGCACTGTGTTA
WT-ARB3+	GTGGTGGCTGAGAATGGACT
WT-ARB3-	GGGGACAGTGTGAGAAGCTC
WT-ARB4+	GGTGCATGACTACAAGGAGATG
WT-ARB4-	GGGCACACCCTGTAATTGAG
WT-ARB5+	TGAAGCCATTAGGTCCCAAG
WT-ARB5-	ATGGGATCACCGTGTCTTTC
WT-ARB6+	GCTCAGACTGGGATCAAGGA
WT-ARB6-	CCTGGCAGTTTCCTCATAGC
WT-ARB7+	TGCTGCTGACCTCAAGTCC
WT-ARB7-	GAAGAGAACAACGTGCACCA

WT-ARB8+	CGAAAAAGCACAGTGAGCAG
WT-ARB8-	AGGCCTTTCAAATAGTCCATGA
WT-ARB9+	ACCTTGGCTACATTGAGACTCC
WT-ARB9-	TGACACAGGGTTCAGACAGC
WT-ARB10+	AGCCTGTGCTTGCTAGCTG
WT-ARB10-	ACACATGCAGGTGACTCAGG
WT-ARB11+	TCCTGGCGTACTGTCATCAA
WT-ARB11-	CACCAAGTTCCCATACAGCA
WT-ARB12+	TCAGCTATATCCAGAGCCTTCC
WT-ARB12-	GGGAACAGGTTGCTCATGTAA
WT-ARB13+	TGTGAAACACTGCCTTCTGG
WT-ARB13-	TCGACCTTGCAGTAACCACA
**qRT-PCR primer name**	**Primer sequence (5' → 3')**
18S mRNA+	AAACGGCTACCACATCCAAG
18S mRNA-	CAATTACAGGGCCTCGAAAG
Acat2 mRNA+	GGAGTAGGACCAATTCCAGCC
Acat2 mRNA-	GGAGTAGGACCAATTCCAGCC
Acox3 mRNA+	GCCTATGCCTTGGACCACTT
Acox3 mRNA-	ATCTCACGTCCGAGTTCTGC
Arrdc2 mRNA+	ATAAACACACCTGCCCTGCT
Arrdc2 mRNA-	AGTGTAGCCTTTGCGGTCAA
Calml3 mRNA+	ACTGACAGCGAGGAGGAGAT
Calml3 mRNA-	ACTGACAGCGAGGAGGAGAT
Ccno mRNA+	AATACGGCCAGAGCTGCTAC
Ccno mRNA-	AGCCAGCTGAGCAGTTTACA
Creld2 mRNA+	ATCCAGTGTGAAGTGGGCTG
Creld2 mRNA-	CCCACGCAGGTAGAATCACA
Cyp4f15 mRNA+	GCCCGTCTGGATGTGTTTGA
Cyp4f15 mRNA-	ACCGTTTGGTCACTAAGGCA
Ehd3 mRNA+	GGTGGTGGATCCAAAGAAACC
Ehd3 mRNA-	AGAGAGGATCCCCGGTGTG
Fkbp5 mRNA+	TGAGCTCCTTGATTTCAAAGGTGAGG
Fkbp5 mRNA-	CCACAGCAGCCTTCCAGGTGG
Isyna1 mRNA+	CGGCCCTCAGTCTACATTCC
Isyna1 mRNA-	CCGCACTGGATCGGAAATCT
Kcnk1 mRNA+	TTCCAGGGGAAGGCTACAAC

Kcnk1 mRNA-	TTCCAGGGGAAGGCTACAAC
Lcn12 mRNA+	AGCCTTCTGGGTAGAAACTGG
Lcn12 mRNA-	TCCTTCTTCAGCAGACTTGTGG
Lipg mRNA+	TATCACAACCTTGCGTCCCC
Lipg mRNA-	CTGGGTCCTTAGAAGTGCGG
Pdk2l1 mRNA+	CCTGGGCCCTGTGTACTTTG
Pdk2l1 mRNA-	TTGACCTCGGAGTATGTGTCG
Ramp3 mRNA+	TCTGGAAGTGGTGCAACCTG
Ramp3 mRNA-	GTCTCCATCTCGGTGCAGTT
Rhbdd3 mRNA+	CACTGGGAAGCAAATCCAGGT
Rhbdd3 mRNA-	CCCAGGCATGCATATTTGGTT
Rhbg mRNA+	ACTGGGGCAACCACAGTAAC
Rhbg mRNA-	AGCCGTACCGCTGTAGAAAG
**Sequences used in *in vitro* assays**	
**Name**	**Sequence (5’ → 3’), DNA strand**
Sh1	CACAGAACGTCCTGTTCCAGAA (-1)
Sh2	AAGAGGACAGTCTGTACTTGCA (-1)
Sh3	TGGAGAACGGAATGTTCCGCAC (1)
SP1	AAAAGTACAGAATGTTCTGTGA (1)
SP2	CGCAGAACATTCTGTTCTCCTC (-1)
SP3	CCTAGAACACCAAGTCACAGCA (-1)
wt1	CTCATTCCACACAGTCCCTGCC (1)
wt2	AGTGGCCCTGTCTGTCCTTAAA (1)
wt3	AGAAGCTCACTCTGTGCCGCAC (-1)
